# Cytokinin Modulates Responses to Phytomelatonin in *Arabidopsis thaliana* under High Light Stress

**DOI:** 10.3390/ijms24010738

**Published:** 2023-01-01

**Authors:** Ivan A. Bychkov, Aleksandra A. Andreeva, Natalia V. Kudryakova, Victor V. Kusnetsov

**Affiliations:** K.A. Timiryazev Institute of Plant Physiology RAS, 35 Botanicheskaya St., Moscow 127276, Russia

**Keywords:** *Arabidopsis thaliana*, cytokinin, gene expression, high light stress, melatonin, mutants

## Abstract

Fine-tuned interactions between melatonin (MT) and hormones affected by environmental inputs are crucial for plant growth. Under high light (HL) conditions, melatonin reduced photodamage in *Arabidopsis thaliana* and contributed to the restoration of the expression of the cytokinin (CK) synthesis genes *IPT3*, *IPT5* and *LOG7* and genes for CK signal transduction *AHK2*,*3* and *ARR 1*, *4*, *5* and *12* which were downregulated by stress. However, CK signaling mutants displayed no significant changes in the expression of CK genes following HL + MT treatment, implying that a fully functional cytokinin signaling pathway is a prerequisite for MT–CK interactions. In turn, cytokinin treatment increased the expression of the key melatonin synthesis gene *ASMT* under both moderate and HL in wild-type plants. This upregulation was further accentuated in the *ipt3*,*5*,*7* mutant which is highly sensitive to CK. In this mutant, in addition to *ASMT*, the melatonin synthesis genes *SNAT* and *COMT*, as well as the putative signaling genes *CAND2* and *GPA1*, displayed elevated transcript levels. The results of the study suggest that melatonin acts synergistically with CK to cope with HL stress through melatonin-associated activation or repression of the respective hormonal genes.

## 1. Introduction

Melatonin (*N*-acetyl-5-methoxytryptamine), a multifunctional biomolecule, mediates many important functions, both in growth stimulation and stress tolerance [1]. The molecular mechanisms linking melatonin with plant hormones are of particular interest due to the hormone-like functions exhibited by melatonin at low concentrations and its direct participation in the modulation of gene expression [2,3]. However, certain antioxidant functions of melatonin at concentrations elevated by orders of magnitude do not exclude interactions with phytohormones. According to current data, melatonin modulates the metabolism of plant hormones (biosynthesis and catabolism), the rise or fall in their endogenous levels, and the regulation of signaling elements, thereby affecting the final responses to auxin, gibberellins, cytokinins, abscisic acid, ethylene, salicylic acid, jasmonates, brassinosteroids, polyamines and strigolactones [4].

One of the well-known effects of melatonin is its participation in abiotic stresses such as heat, cold, drought, toxic chemicals and photodamage. Counteracting these hazards implies cross-reactions with phytohormones which, for their part, can be indispensable participants in these processes. There is increasing evidence that CK has a variety of functions in plant responses to various stresses and in crosstalk with melatonin via modulation of the components of the CK signaling pathway and genes encoding CK metabolism enzymes.

CK synthesis includes the formation of CK nucleotides, which is catalyzed by isopentenyl transferases (IPTs) and the subsequent conversion of the CK nucleotides into the free bases isopentenyladenine (iP) and *trans*-zeatin (tZ) by the activity of the LONELY GUY (LOG) enzymes. The cleavage of CK is catalyzed by CK-oxidase/dehydrogenases (CKXs) [5]. The CK signal is perceived by the two-component pathway involving three histidine kinase receptors (AHK4/CRE1/WOL, AHK2, and AHK3), histidine phosphotransfer proteins (AHPs) and two classes of response regulators acting as transcription factors (type B ARRs) or negative regulators of CK signaling (type A ARRs) [6].

An example of an interaction between the components of the CK signaling pathway and melatonin was presented by Zhang et al. [7]. The alleviation of heat stress by melatonin in perennial ryegrass *(Lolium perenne)* was the result of cross-talk between melatonin and CKs: two CK biosynthesis genes (*LpIPT2* and *LpOGI*) were upregulated by melatonin in stress conditions, while genes for A-ARR and B-ARR transcription factors, involved in CK signaling, were down- and upregulated, respectively. Melatonin also suppressed two ABA signaling transcription factors, *LpABI3* and *LpABI5,* and downregulated two ABA biosynthesis genes, *LpZEP* and *LpNCED*, thereby delaying heat-induced senescence. Generally, an increase in melatonin content can lead to downregulation of ABA-responsive genes and a decrease in ABA-mediated repression of CK-related genes.

CKs and melatonin acted synergistically in wild-type and isopentenyl transferase-overexpressing transgenic creeping bentgrass *Agrostis stolonifera* under drought conditions to increase photochemical efficiency, chlorophyll content and relative water content [8]. Melatonin treatment also activated several CK response regulators, A-ARR and B-ARR, to increase the growth of *B. napus* [9]. In sweet cherry, the endogenous contents of cytokinins were transiently affected by melatonin treatment at 10^−5^ M, in addition to ABA, salicylic acid and jasmonic acid, thereby exerting a modulatory role on fruit ripening through hormonal cross-talk. Recently, the role of CK in cross-interaction with melatonin was further highlighted in walnut: *ASMT* family genes encoding a key enzyme that catalyzes the final stage of melatonin biosynthesis were predicted to be coexpressed with the CK synthesis gene *LOG* [10].

To approximate the putative mechanisms of cross-reactions between melatonin and CKs, we examined the role of melatonin in modulating the expression of CK-associated genes under photoinhibitory conditions as such an interaction may be especially relevant to photoprotection. Recent research has provided evidence that melatonin was transiently induced in response to HL stress in *Arabidopsis thaliana* with a simultaneous increase in the expression of melatonin biosynthetic genes [11]. On the other hand, the role of CKs as contributors to stress tolerance [12,13,14,15,16,17], especially to light stress [18], suggests their possible regulatory functions in maintaining melatonin metabolic and/or signaling pathways. Therefore, the main goal was focused on the search for a regulatory interaction of melatonin with cytokinin. The results of studying CK mutants indicate that melatonin may act synergistically with CK via melatonin-mediated activation or repression of the CK synthesis and signaling genes. Reciprocally, CK is involved in expression regulation of the genes for melatonin metabolism and signal transduction, with the melatonin biosynthesis gene *ASMT* as a key component in the crosstalk between CK and MT metabolic pathways.

## 2. Results

### 2.1. Effect of HL Stress and Exogenous Melatonin on Photosynthetic Activity and Transcript Abundance of Chloroplast Genes in Mutants with Reduced Cytokinin Status

Mutants with a reduced CK status generally exhibit a strong stress-tolerant phenotype that is associated with increased cell membrane integrity and ABA hypersensitivity and altered expression of more than 10% of the annotated *Arabidopsis* genes [15,16]. In addition, they were also shown to be more susceptible to severe HL stress (1000 μmol m^−2^ s^−1^, 24 h) and exhibited reduced efficiency of photoprotective mechanisms [18], indicating the dependence of the response on the magnitude of stress. To test whether there is cross-talk between melatonin and CK under HL, we focused on the triple CK-deficient *ipt3*,*5*,*7* mutant, the CK receptor double mutant *ahk2*,*3* and the triple mutant for B-type ARR genes *arr1*,*10*,*12* as the respective genes regulate the majority of cytokinin effects during vegetative development [19,20].

**Figure 1:**
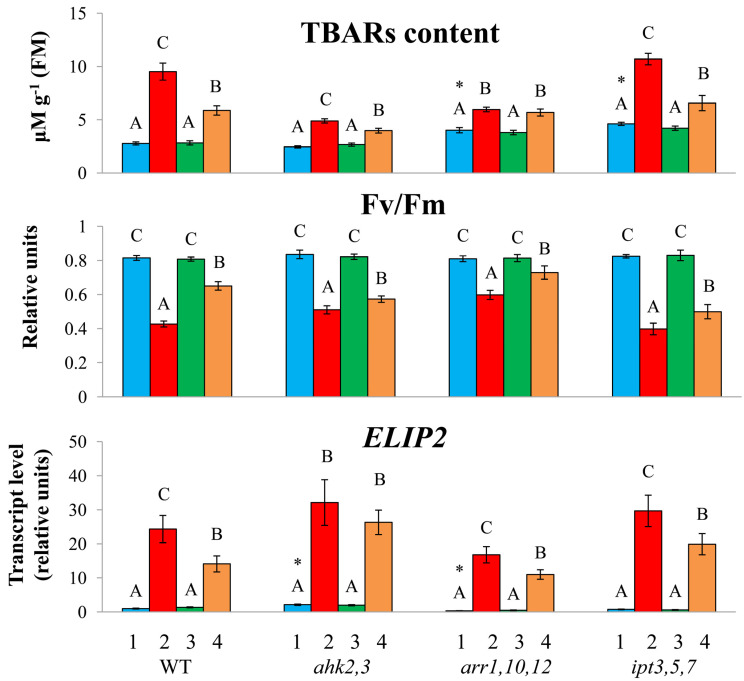
Effect of HL stress and melatonin treatment on the TBARs content, Fv/Fm parameter and expression of the *ELIP2* gene. The data presented in the figure are the mean values (*n* ≥ 3). The error bars represent SEs. Different letters denote statistically significant differences among variants within the same genotype at *p* < 0.05 (ANOVA with post hoc Tukey’s multiple-comparison test). Asterisks indicate statistically significant differences between the mutants and the wild-type under the corresponding type of treatment at *p* < 0.05 (*t* test).

WT plants challenged with HL stress (600 μmol m^−2^ s^−1^ for 24 h) experienced severe physiological damage, which was accompanied by a decrease in melatonin concentration and downregulation of the genes for melatonin biosynthesis [21]. According to our results, in the mutants with a reduced cytokinin status, HL increased lipid peroxidation and triggered a strong upregulation of an indicator of light stress *ELIP2 (EARLY LIGHT-INDUCIBLE PROTEIN 2)* which encodes a protein with photoprotective functions [22]. Induction levels were highest in *ipt3*,*5*,*7* and *arr1*,*10*,*12* with reduced steady state expression levels and lowest in *ahk2*,*3*, with constitutively elevated levels of *ELIP2* (Figure 1). The photochemical efficiency of PSII (Fv/Fm) was reduced under high irradiance, although the reduction was not stronger in the mutants than in the wild-type. The melatonin treatment decreased photodamage in wild-type plants and to varying degrees in mutant plants.

**Figure 2:**
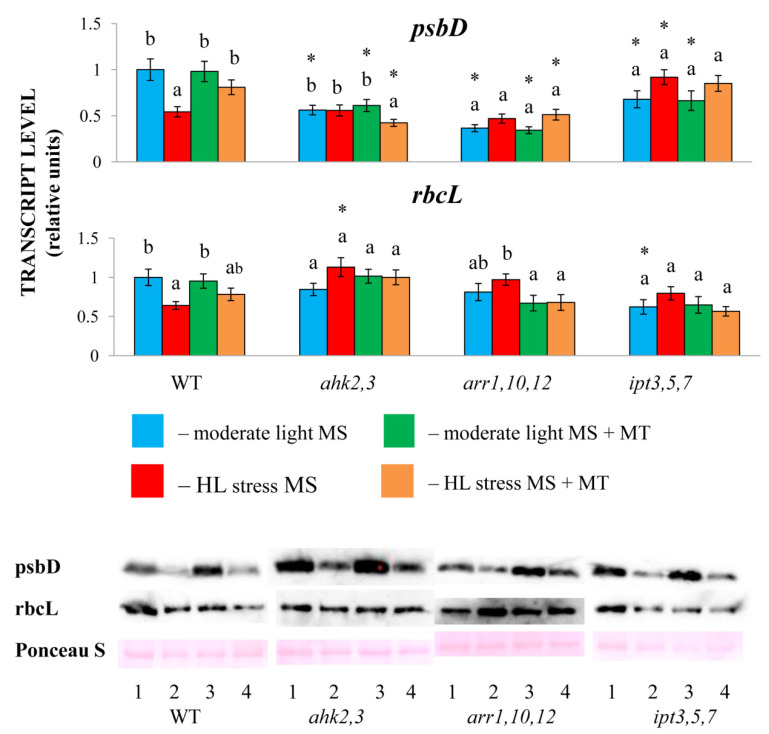
Effect of HL stress and melatonin treatment on the expression of chloroplast genes and corresponding proteins. 1—moderate light MS; 2—HL stress MS; 3—moderate light MS + melatonin; 4—HL stress MS + melatonin. The data presented in the figure are the mean values (*n* ≥ 3). The error bars represent SEs. Different letters denote statistically significant differences among variants within the same genotype at *p* < 0.05 (ANOVA with post hoc Tukey’s multiple-comparison test). Asterisks indicate statistically significant differences between the mutants and the wild-type under the corresponding type of treatment at *p* < 0.05 (*t* test).

The fact that the mutants did not experience a higher degree of photodamage compared with the WT was further confirmed by the maintenance of control transcript levels of the selected chloroplast genes *psbD* (*PSII*) and *RbcL* (Rubisco), albeit they were expressed at lower basal levels in the mutants (Figure 2). This was combined with the absence of a reliable increase in transcript accumulation in response to MT treatment. However, the protein levels of the reaction center protein D2 decreased upon HL stress, although to a lesser extent than in the WT and were partly restored following MT treatment (Figure 2). The discrepancy between the level of the D2 protein and the content of their mRNA suggests that the regulation of protein content occurs largely at the posttranscriptional level, which may involve impaired mechanisms of mRNA processing and cotranslational modifications similar to those described for D1 protein [18,23] and/or impaired de novo synthesis. At the same time we did not find any differences in the protein content of RBCL in the wild-type or the mutants under all modes of treatment (Figure 2), which points to a balance between photodamage and recovery from photoinhibition upon HL. It should be noted that in our previous work (21), approximately identical protein levels were also found for atpB (ATP synthase) and psaB (PSI), although the corresponding transcript levels were strongly regulated by HL and melatonin.

### 2.2. Melatonin Alters Endogenous Melatonin Content and the Expression of Melatonin Synthesis Genes in CK Mutants under High Irradiance

**Figure 3:**
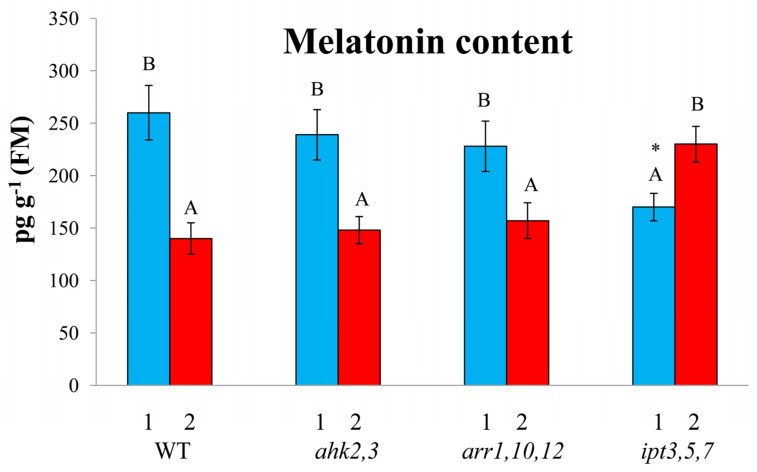
Effect of HL stress on the melatonin content. The data presented in the figure are the mean values (*n* ≥ 3). Error bars represent SEs. Different letters denote statistically significant differences between variants within the same genotype at *p* < 0.05 (ANOVA with post hoc Tukey’s multiple-comparison test). Asterisks indicate statistically significant differences between the mutants and the wild-type under the corresponding type of treatment at *p* < 0.05 (*t* test).

**Figure 4:**
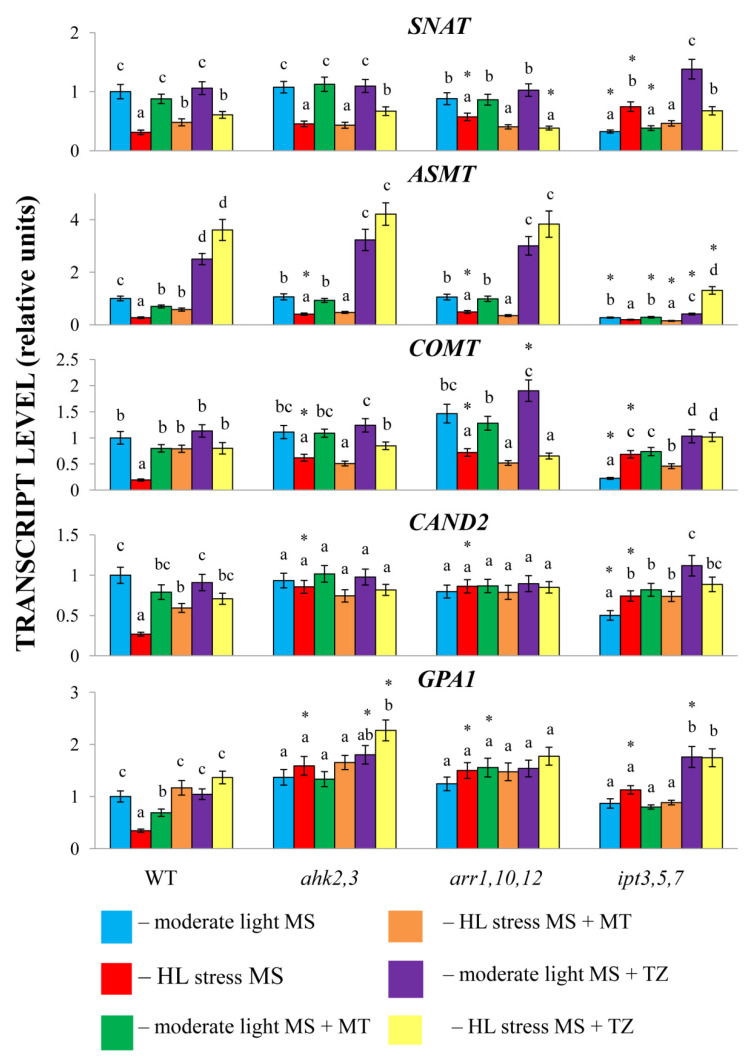
Effect of HL stress and melatonin and *trans*-zeatin treatment on the expression of melatonin synthesis and signaling genes. The data presented in the figure are the mean values (*n* ≥ 3). Error bars represent SEs. Different letters denote statistically significant differences among variants within the same genotype at *p* < 0.05 (ANOVA with post hoc Tukey’s multiple-comparison test). Asterisks indicate statistically significant differences between the mutants and the wild-type under the corresponding type of treatment at *p* < 0.05 (*t* test).

Under both moderate and high light, the melatonin content in CK signaling mutants (*ahk2*,*3*, *arr1*,*10*,*12*) was comparable to that of WT, as was their expression levels of MT biosynthesis genes *SNAT* (serotonin *N*-acetyltransferase), *ASMT* (*N*-acetylserotonin methyltransferase), and *COMT* (caffeic acid *O*-methyltransferase) (Figure 3 and Figure 4). In contrast, the *ipt3*,*5*,*7* mutant had a reduced steady-state melatonin content and a reduced expression of MT synthesis genes. Under high irradiance, the melatonin content and the expression of *SNAT* and *COMT* increased in *ipt3*,*5*,*7*, which was similar to the results obtained by Lee and Back [11] for WT and MT mutants under less prolonged stress treatment (600 μmol m^−2^ s^−1^ for 6 h). Such a reaction was probably explained by the increased stress resistance of the *ipt3*,*5*,*7* mutant deficient for CK synthesis which allowed it to maintain a higher level of MT synthesis under severe light stress, whereas it was already inhibited in wild-type plants.

Treatment with exogenous MT under moderate light did not significantly alter the transcript abundance of MT synthesis or signaling genes in CK mutants, except for a three-fold increase in *COMT* transcript levels in the *ipt3*,*5*,*7* mutant. Likewise, treatment with MT under high irradiance did not contribute to a significant increase in the expression levels of MT synthesis genes in the CK signaling mutants *ahk2*,*3* and *arr1*,*10*,*12* as was the case with wild-type plants. In addition, the accumulation of *CAND2* transcripts remained amazingly stable in the CK signaling mutants, although in WT plants, their levels were significantly downregulated by stress and partly restored under simultaneous exposure to HL and melatonin. At the same time, transcript levels of the putative melatonin receptor *CAND2* were significantly increased in the *ipt3*,*5*,*7* mutant under stress and/or melatonin treatment. Although the validity of CAND2 as a receptor remains controversial, it may play a specific role in plants by binding melatonin, which can reduce free melatonin levels [1]. In any case, the nonwild-type regulation of *CAND2* and MT synthesis genes in CK mutants suggests the necessity of operating the CK signaling chain for transcriptional regulation of MT genes and the existence of a fine-tuning mechanism that maintains the appropriate MT:CK ratio.

In summary, it can be concluded that cytokinin deficiency and disruption of the CK signaling pathway affect the metabolism and protective functions of melatonin in the cell.

### 2.3. Melatonin Regulates CK-Associated Genes under Photoinhibitory Conditions

**Figure 5:**
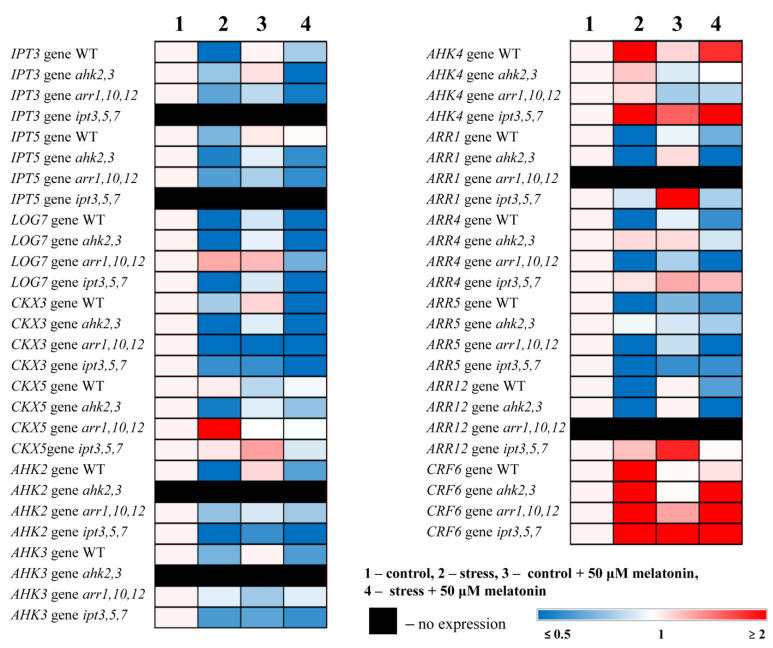
Effect of HL stress and melatonin on the expression of cytokinin metabolism and signaling genes. Numerical data are presented in Table S1.

To gain further insight into the interplay between MT and CK-associated genes, we examined the effect of MT and stress treatment on the expression levels of hormone-related genes in CK mutants. It is believed that the cytokinin dependent light stress responses are mainly mediated by the cytokinin receptor AHK3 and to a lesser extent by AHK2 and the B-type response regulators ARR1 and ARR12 since *ahk2*,*3* and *arr1*,*12* double mutants were shown to be hypersensitive to high-light stress [18]. Nevertheless, the expression of the genes for AHK2 and AHK3 CK receptors and the type B response regulators ARR1 and ARR12 were generally downregulated in WT and CK signaling mutants under HL, and the expression of the type A response regulators ARR4 and ARR5 was also down-regulated, supporting a negative regulatory role for CKs in stress tolerance [15]. In accordance with this concept, high light downregulated the CK synthesis genes *IPT3*, *IPT5*, and *LOG7* (encodes CK-activating enzyme operating in the final step of bioactive CK synthesis), as well as the CK catabolizing gene *CKX3*, but did not change the expression of *CKX5* (Figure 5, Table S1). However, *AHK4* was upregulated by HL stress in WT and *ipt3*,*5*,*7* and remained stable in *ahk2,3* and *arr1,10,12*, suggesting that this receptor might have specific functions in HL stress responses.

When exposed to MT + HL, the levels of CK-associated gene transcripts were generally less suppressed in the WT. The expression of the Cytokinin Response Factor *CRF6*, which acts as a transcriptional repressor during oxidative stresses [24], returned to its control values in the WT after a two-fold increase under HL, and the expression of *AHK4* decreased by half compared with the stress level. This may be the result of a more stable state of plants treated with melatonin in which the stress-induced response is mitigated. Melatonin also significantly changed the expression of CK signaling genes in the cytokinin-deficient *ipt3*,*5*,*7* mutant, even under moderate illumination (i.e., suppressed *AHK2*, *AHK3* and *ARR5* but upregulated *AHK4*, *ARR1*, *ARR12* and *CRF6*), and strongly inhibited *CKX3.* Taking into account that the endogenous melatonin level in *ipt3*,*5*,*7* was reduced by approximately one-third of the wild-type level, these results suggest that CK-deficient plants might possess an improved ability to sense and/or respond to extracellular melatonin to compensate for the reduction in endogenous MT levels. In addition, the CK signaling mutants displayed no significant changes in the expression of CK genes following HL + melatonin treatment, implying that a fully functional cytokinin signaling pathway is a prerequisite for MT–CK interactions.

### 2.4. CK Treatment Causes a Differential Response of MT-Related Genes in Hormonal Mutants

Subsequently, we investigated the response of MT biosynthesis genes to CK treatment. Exogenous cytokinin increased the *ASMT* transcript levels under moderate light in the wild-type and unexpectedly in all CK mutants including *ahk2*,*3* and *arr1*,*10*,*12* (Figure 4). Upregulation of *ASMT* by CK in the mutants with strongly reduced cytokinin signaling activity indicates that a compensatory mechanism may be activated under conditions of partial cytokinin insensitivity. Regulation of melatonin gene expression by CK was further accentuated in the *ipt3*,*5*,*7* mutant where, in addition to *ASMT*, *SNAT* and *COMT* as well as *CAND2* and *GPA1* displayed elevated transcript levels. The altered sensitivity of MT metabolic genes in CK mutants is consistent with the primary role of *ASMT* in CK-dependent induction of melatonin synthesis, followed by secondary roles for *SNAT* and *COMT.*

HL exposure in combination with CK treatment resulted in more than a three-fold upregulation of the *ASMT* gene in highly CK-sensitive *ipt3*,*5*,*7* and further activated *ASMT* expression in WT. Under stress conditions, cytokinin also retarded the decrease in the expression of *SNAT* and *COMT* in the wild-type and slightly in *ahk2*,*3* but it did not affect these genes in *arr1*,*10*,*12*. Collectively, these results indicate that *CK* may act synergistically with melatonin in the regulation of MT synthesis genes under both normal and stress conditions.

## 3. Discussion

The concept that melatonin is a phytohormone defines the necessity to clarify its possible functions in the phytohormone network. The dominant role played by cytokinins in many aspects of plant biology makes them prime candidates for crosstalk with melatonin, including the regulation of stress responses, which can be achieved through direct or indirect interactions between signaling and metabolic pathways. Despite the fact that the individual contribution of melatonin to stress tolerance, as well as that of CK, has been extensively explored, less attention has been given to their combined influence. Here, we demonstrate that melatonin effects were intimately linked to CK signaling and metabolic circuits. Under HL stress, exogenous melatonin upregulated CK-associated genes. In this case, the effect of melatonin can be interpreted in terms of stress mitigation. Melatonin increased plant viability, which resulted in the activation of CK genes that are commonly/predominantly suppressed in response to oxidative stress. In turn, the levels of MT synthesis gene transcripts increased in response to CK treatment, even under moderate illumination. However, this regulation was disrupted in hormonal mutants. Therefore, melatonin, at least in part, acts synergistically with CK. These data are consistent with the results of the study of the interaction between melatonin and cytokinin in *Agrostis stolonifera* under drought conditions and in *B. napus* [8,9].

The diversity and complexity of the CK action may also include their direct participation in stress protective mechanisms. Both exogenous application of CKs and the employment of transgenic plants with enhanced CK production improved the antioxidant capacity and secondary metabolite content [25]. Moreover, AHK2 and AHK3, as well as type B ARR 1 and ARR12, were implicated in the routine repair of D1 protein during light stress, which is necessary for the functioning of PSII [18]. According to our results, melatonin treatment also maintained the expression of *psbD* and in a portion of the corresponding proteins at a higher level in the stressed WT and mutants compared with untreated plants, although distinct transcriptional induction of *psbD* was not observed in CK mutants. These findings raise important questions about the underlying mechanisms by which melatonin and CK provoke similar biological outcomes. In addition to shared transcriptional targets, such a response suggests that the long-term effects of treatments with various hormones may represent a ‘domino effect’ that resets many systems within the plant [26].

A more detailed analysis of *ipt357*, which is highly sensitive to CK treatment, indicated that, in addition to *ASMT*, *SNAT* and *COMT* as well as the putative MT receptor genes *CAND2* and *GPA1* may be upregulated by CK, both under normal and high irradiance. Moreover, melatonin treatment of *ipt357* under moderate and excessive light also contributed to an increase in MT transcripts. Activation of extra participants of MT-related pathways in a context-specific manner may provide a means to fine-tune the MT response to multiple challenges given that the activity of their gene products is dosage sensitive.

The *ipt3*,*5*,*7* mutant, with its improved ability to sense extracellular melatonin, provides special opportunities for assessing the effect of melatonin treatment on CK-associated genes. Even under moderate light, melatonin suppressed *CKX3* and *ARR5* but had no significant impact on *CKX5* or *ARR4* which are both expected to perform similar functions. Melatonin downregulated *AHK2* (and to a lesser extent *AHK3*) in the *ipt3*,*5*,*7* mutant under normal light and especially under excess light and upregulated *AHK4*. This is consistent with the notion that distinct members of protein families can form highly specialized nonoverlapping regulatory modules under the influence of specific effectors. Interestingly, in contrast to AHK3, AHK2 was shown to be a positive regulator of aging either directly or indirectly associated with a specific set of genes involved in the regulation of iron efflux and degradation of cell wall components at the final stages of leaf ontogenesis [27,28]. It is tempting to speculate that AHK2 may be a key component of the crosstalk between CK and MT circuits while contributing to senescence-retarding effects.

According to our results, melatonin treatment of the CK loss-of function signaling mutants under high irradiance did not alter the transcript levels of the MT signaling and synthesis genes, which suggests the necessity of a fully functional signaling pathway. Another possibility is that the effects of the altered hormonal status mimic the action of exogenous melatonin since mutants originally identified as defective in one response may show defects in other responses. For example, the response to MT treatment may be near saturation when taking into account the elevated CK content in the CK signaling mutants [29] and the synergistic effect of MT and CK action.

Much more surprising was the fact that exogenous cytokinin increased the *ASMT* transcript levels in the CK loss-of-function signaling mutants. This ambiguity could be explained by the high functional redundancy of B-type ARRs and CK receptors, possibly indicating that the examined genes were not obligatory players in mediating cytokinin action in this context. This probably suggests the involvement of the AHK4 receptor and additional ARR-B or CRF transcription factors able to mediate cytokinin responses independent of ARRs. In addition, the highly interconnected web involved in hormone metabolism and signaling suggests the emergence of compensatory mechanisms generating alternative routes for the CK-induced activation of melatonin synthesis. Recently, it was shown that a large majority of signaling proteins function pleiotropically in several pathways contributing to phytohormone signal integration [30]. Further studies involving global transcriptomic analysis are needed to fully investigate the regulatory networks underlying the crosstalk between melatonin and CKs.

Our finding that CK modulates *ASMT* expression provides a link between melatonin and this hormone and indicates that ASMT may act as an important crosstalk hub between MT and CK. In silico analysis using the AthaMap server http://www.athamap.de/, accessed on 29 December 2022, revealed among the cis-regulatory elements found approximately within 500 bp upstream of the transcription start site several type B ARRs consensus binding sites for ARR11 and ARR14 and binding elements for GLK1 and GATA12 transcription factors that can be engaged in CK-dependent regulation of ASMT expression. However, more work will be required to prove physical interaction between these transcription factors and ASMT. Notably, one of them, ARR11, belongs to the CK-associated module of CRF6-dependent genes engaged in oxidative stress responses [24].

## 4. Materials and Methods

### 4.1. Plant Material, Growth, and Treatment Conditions

T-DNA insertion mutants (all ecotype Col-0) were obtained from the Nottingham Arabidopsis Stock Centre or kindly provided by Prof. Tatsuo Kakimoto from Osaka University, Japan. The seeds were stratified for 48 h at 4 °C in the dark and germinated in half strength Murashige and Skoog (MS) medium with 1% sucrose and 0.5% agar in a growth chamber at 23 °C with a 16-h photoperiod and photosynthetic photon flux density of 60 μmol m^−2^ s^−1^. At the age of 2 weeks, the seedlings were pretreated with 50 μM of melatonin or 5 μM of *trans*-zeatin for 72 h and were either left under the same growing conditions or placed for 24 h under the HPI-T2 2000 W/646 lamp (Philips, Netherlands) with a luminous energy flux of 600 μmol m^−2^ s^−1^. The concentrations of active reagents and treatment time were selected in preliminary experiments (Table S3). A long pretreatment period (three days before the onset of stress) was chosen since melatonin is believed to have an amphiphilic nature [31], which implies a relatively low penetrating ability. A combination of air and water cooling was used to minimize the thermal impact of the lamp. At the end of the exposure, measurements were directly taken or the samples were frozen in liquid nitrogen and stored at −80 °C.

### 4.2. Fluorometry

The maximal photochemical efficiency of PSII Fv/Fm was determined with a DUAL-PAM101 (Walz, Germany) in accordance with Kozuleva et al. [32] after dark adaptation for 30 min. The following parameters were determined: measuring light, 460 nm, 9 µmol m^−2^ s^−1^; saturating pulses, 500 ms, 635 nm, 4000 µmol m^−2^ s^−1^; actinic light, 635 nm, 37 µmol m^−2^ s^−1^. The actinic light duration was 240 s.

### 4.3. Determination of TBARS

The level of TBARs (secondary products of the lipid peroxidation of membranes that react with thiobarbituric acid) was measured as described by Heath and Packer [33].

### 4.4. Melatonin Measurement

Melatonin measurement was performed as described by Lee and Back [11] using ELISA Kit CEA908GE (Cloud-Clone Corp., Katy, TX, USA) in accordance with the manufacturer’s instructions. The optical density was measured at 450 nm with Multiskan MS Microplate Reader LabSystems 352 (Thermo/LabSystems, Pittsburg, PA, USA).

### 4.5. RNA Isolation and Quantitative Real Time (RT)-PCR

The expression levels of nuclear and chloroplast genes were evaluated by RT—PCR according to Danilova et al. [34] in a LightCycler 96 (Roche, Rotkreuz, Switzerland). The nucleotide sequences of the primers for quantitative RT—PCR analyses are presented in Table S2. The following standard thermal profile was used for all PCR reactions: 95 °C for 5 min, 40 cycles of 95 °C for 15 s, 58 °C for 15 s and 72 °C for 25 s. All data were normalized to the transcript levels of the nuclear-encoded polyubiquitin *UBQ10* gene which was used as an internal control.

### 4.6. Protein Gel-Blot Analysis

Protein extraction and gel-blot analysis were performed as described previously [35]. The samples were blotted onto PVDF membranes after SDS—PAGE electrophoresis, and the blots were incubated with anti-PsbD (PSII; AS06 146) and anti-RbcL (Rubisco; AS03 037 primary antibodies (Agrisera, Vännäs, Sweden) overnight at 4 °C followed by the secondary antibody (anti-rabbit IgG horse radish peroxidases conjugated from Agrisera, AS09 602) for 1 h at room temperature according to the manufacturer’s instructions. Signals from immunoblotting were detected using the ECL method (ECL Western Blotting Detection Kit, Bio-Rad, Hercules, CA, USA) by the Invitrogen iBright Imaging Systems (Thermo Fisher Scientific, Pittsburg, PA, USA).

### 4.7. Statistical Data Processing

All the experiments were performed using at least three biological replicates. For statistical analyses of physiological parameters, we used SigmaPlot 12.3 (Systat Software Inc., Richmond, California, USA) with one-way analysis of variance (ANOVA) followed by Duncan’s method. Statistical analyses of gene expression data were performed with ANOVA with post hoc Holm multiple-comparison calculation using the online calculator (astatsa.com/OneWay_Anova_with_TukeyHSD/). All data are presented as the mean values ± standard errors (SEs).

## 5. Conclusions

In summary, it can be concluded that melatonin, as a biostimulator, may modulate the HL stress response by regulating the expression of CK synthesis and signaling genes as a part of genome-wide transcriptional reprogramming. On the other hand, cytokinin deficiency and disruption of the CK signaling pathway affect the metabolism and protective functions of melatonin in the cell. Based on the research of the CK mutants, we hypothesized that melatonin acts synergistically with CK to cope with HL stress through melatonin-associated activation or repression of the respective hormonal genes. The altered sensitivity of MT metabolic genes in CK mutants is consistent with the primary role of *ASMT* in the CK-dependent induction of melatonin synthesis, followed by secondary roles for *SNAT* and *COMT.*

## Supplementary Materials

The following supporting information can be downloaded at: https://www.mdpi.com/article/10.3390/ijms24010738/s1.

## References

[1] Back K. (2021). Melatonin metabolism, signaling and possible roles in plants. Plant J..

[2] Hardeland R. (2016). Melatonin in plants-diversity of levels and multiplicity of functions. Front. Plant Sci..

[3] Arnao M.B., Hernández-Ruiz J. (2018). Melatonin and its relationship to plant hormones. Ann. Bot..

[4] Arnao M.B., Hernández-Ruiz J. (2021). Melatonin as a regulatory hub of plant hormone levels and action in stress situations. Plant Biol..

[5] Cortleven A., Leuendorf J.E., Frank M., Pezzetta D., Bolt S., Schmülling T. (2019). Cytokinin action in response to abiotic and biotic stresses in plants. Plant Cell Env..

[6] Kieber J.J., Schaller G.E. (2018). Cytokinin signaling in plant development. Development.

[7] Zhang J., Shi Y., Zhang X., Du H., Xu B., Huang B. (2017). Melatonin suppression of heat-induced leaf senescence involves changes in abscisic acid and cytokinin biosynthesis and signaling pathways in perennial ryegrass (*Lolium perenne* L.). Env. Exp. Bot..

[8] Ma X., Zhang J., Burgess P., Rossi S., Huang B. (2018). Interactive effects of melatonin and cytokinin on alleviating drought-induced leaf senescence in creeping bentgrass (*Agrostis stolonifera*). Env. Exp. Bot..

[9] Tan X., Long W., Zeng L., Ding X., Cheng Y., Zhang X., Zou X. (2019). Melatonin-induced transcriptome variation of rapeseed seedlings under salt stress. Int. J. Mol. Sci..

[10] Ma K., Xu R., Zhao Y., Han L., Xu Y., Li L., Wang J., Li N., Walnut N. (2022). Acetylserotonin methyltransferase gene family genome-wide identification and diverse functions characterization during flower bud development. Front. Plant Sci..

[11] Lee H.Y., Back K. (2018). Melatonin induction and its role in high light stress tolerance in Arabidopsis thaliana. J. Pineal Res..

[12] Rivero R.M., Kojima M., Gepstein A., Sakakibara H., Mittler R., Gepstein S., Blumwald E. (2007). Delayed leaf senescence induces extreme drought tolerance in a flowering plant. Proc. Natl. Acad. Sci. USA.

[13] Tran L.S., Urao T., Qin F., Maruyama K., Kakimoto T., Shinozaki K., Yamaguchi-Shinozaki K. (2007). Functional analysis of AHK1/ATHK1 and cytokinin receptor histidine kinases in response to abscisic acid, drought, and salt stress in Arabidopsis. Proc. Natl. Acad. Sci. USA.

[14] Jeon J., Kim N.Y., Kim S., Kang N.Y., Novák O., Ku S.J., Kim J. (2010). A subset of cytokinin two-component signaling system plays a role in cold temperature stress response in *Arabidopsis*. J. Biol. Chem..

[15] Nishiyama R., Watanabe Y., Fujita Y., Le D.T., Kojima M., Werner T., Vankova R., Yamaguchi-Shinozaki K., Shinozaki K., Kakimoto T. (2011). Analysis of cytokinin mutants and regulation of cytokinin metabolic genes reveals important regulatory roles of cytokinins in drought, salt and abscisic acid responses, and abscisic acid biosynthesis. Plant Cell..

[16] Nishiyama R., Le D.T., Watanabe Y., Matsui A., Tanaka M., Seki M., Tran L.S. (2012). Transcriptome analyses of a salt-tolerant cytokinin deficient mutant reveal differential regulation of salt stress response by cytokinin deficiency. PLoS ONE.

[17] Macková H., Hronková M., Dobrá J., Turečková V., Novák O., Lubovská Z., Vanková R. (2013). Enhanced drought and heat stress tolerance of tobacco plants with ectopically enhanced cytokinin oxidase/dehydrogenase gene expression. J. Exp. Bot..

[18] Cortleven A., Nitschke S., Klaumünzer M., Abdelgawad H., Asard H., Grimm B., Riefler M., Schmülling T. (2014). A novel protective function for cytokinin in the light stress response is mediated by the Arabidopsis histidine kinase2 and Arabidopsis histidine kinase3 receptors. Plant Physiol..

[19] Ishida K., Yamashino T., Yokoyama A., Mizuno T. (2008). Three type-B response regulators, ARR1, ARR10 and ARR12, play essential but redundant roles in cytokinin signal transduction throughout the life cycle of Arabidopsis thaliana. Plant Cell Physiol..

[20] Cortleven A., Marg I., Yamburenko M.V., Schlicke H., Hill K., Grimm B., Schaller G.E., Schmülling T. (2016). Cytokinin regulates the etioplast-chloroplast transition through the two-component signaling system and activation of chloroplast-related genes. Plant Physiol..

[21] Bychkov I.A., Kudryakova N.V., Pojidaeva E.S., Kusnetsov V.V. (2021). The melatonin receptor CAND2 is involved in the regulation of photosynthesis and chloroplast gene expression in *Arabidopsis thaliana* under photooxidative stress. Photosynthetica.

[22] Tzvetkova-Chevolleau T., Franck F., Alawady A.E., Dall’Osto L., Carrière F., Bassi R., Grimm B., Nussaume L., Havaux M. (2007). The light stress-induced protein ELIP2 is a regulator of chlorophyll synthesis in *Arabidopsis thaliana*. Plant J..

[23] Mulo P., Sakurai I., Aro E.M. (2012). Strategies for *psbA* gene expression in cyanobacteria, green algae and higher plants: From transcription to PSII repair. BBA—Bioenerg..

[24] Zwack P.J., De Clercq I., Howton T.C., Hallmark H.T., Hurny A., Keshishian E.A., Parish A.M., Benkova E., Mukhtar M.S., Van Breusegem F. (2016). Cytokinin response factor 6 represses cytokinin-associated genes during oxidative stress. Plant Physiol..

[25] Hönig M., Plíhalová L., Husičková A., Nisler J., Doležal K. (2018). Role of cytokinins in senescence, antioxidant defence and photosynthesis. Int. J. Mol. Sci..

[26] Nemhauser J.L., Hong F., Chory J. (2006). Different plant hormones regulate similar processes through largely nonoverlapping transcriptional responses. Cell.

[27] Danilova M.N., Kudryakova N.V., Doroshenko A.S., Zabrodin D.A., Rakhmankulova Z.F., Oelmüller R., Kusnetsov V.V. (2017). Opposite roles of the Arabidopsis cytokinin receptors AHK2 and AHK3 in the expression of plastid genes and genes for the plastid transcriptional machinery during senescence. Plant Mol. Biol..

[28] Kudryakova N.V., Danilova M.N., Andreeva A.A., Doroshenko A.S., Klepikova A.V., Shtratnikova V.Y., Kusnetsov V.V. (2021). Inactivation of the cytokinin membrane receptor AHK2 gene causes differential expression of genes of trans-factors involved in regulation of senescence in arabidopsis thaliana. Rus. J. Plant Physiol..

[29] Riefler M., Novak O., Strnad M., Schmulling T. (2006). Arabidopsis cytokinin receptor mutants reveal functions in shoot growth, leaf senescence, seed size, germination, root development, and cytokinin metabolism. Plant Cell..

[30] Altmann M., Altmann S., Rodriguez P.A., Weller B., Elorduy Vergara L., Palme J., Marin-de la Rosa N., Sauer M., Wenig M., Villaecija-Aguilar J.A. (2020). Extensive signal integration by the phytohormone protein network. Nature.

[31] Cipolla-Neto J., Amaral F.G.D. (2018). Melatonin as a hormone: New physiological and clinical insights. Endoc. Rev..

[32] Kozuleva M.A., Lysenko E.A., Klaus A.A., Kuznetsov V.V. (2017). Long-term hyperthermia impairs activity of both photosystems. Dokl. Biochem. Biophys..

[33] Heath L.R., Packer L. (1968). Photoperoxidation in isolated chloroplasts. Kinetics and stoichiometry of fatty acid peroxidation. Arch. Biochem. Biophys..

[34] Danilova M.N., Kudryakova N.V., Voronin P.Y., Oelmuller R., Kusnetsov V.V., Kulaeva O.N. (2014). Membrane receptors of cytokinin and their regulatory role in *Arabidopsis thaliana* plant response to photooxidative stress under conditions of water deficit. Russ. J. Plant Physiol..

[35] Bychkov I.A., Kudryakova N.V., Andreeva A.A., Pojidaeva E.S., Kusnetsov V.V. (2019). Melatonin modifies the expression of the genes for nuclear- and plastid-encoded chloroplast proteins in detached *Arabidopsis* leaves exposed to photooxidative stress. Plant Physiol. Biochem..

