# Supplementary material for: Cytokinin Modulates Responses to Phytomelatonin in Arabidopsis thaliana under High Light Stress
Source: Int J Mol Sci. 2023 Jan 1;24(1):738. doi: 10.3390/ijms24010738 (PMC9821067; doi:10.3390/ijms24010738)
Supplement: Supplementary file 1 [file ijms-24-00738-s001.zip › Table S1.pdf]

Table S1. Effect of HL stress and melatonin on the expression of cytocinin synthesis and signaling genes metabolism, reception and signaling genes. The data presented in the table are the mean values (n ≥ 3). Error bars represent SEs. Different letters denote statistically significant differences between variants within the same genotype at $p < 0.05$ (ANOVA with post hoc Tukey's multiple-comparison test), asterisks indicate statistically significant differences between the mutants and the wild type under corresponding type of treatment at $p < 0.05$ (t-test).

Gene	Genotype	Experimental conditions			
		Control	Stress	Control + 50 μM melatonin	Stress + 50 μM melatonin
IPT3	WT	1.000±0.098^{b}	0.408±0.053^{a}	0.990±0.101^{b}	0.795±0.084^{b}
	ahk2,3	6.787±0.707^{b*}	5.239±0.564^{a*}	7.229±0.809^{b*}	3.427±0.311^{a*}
	arr1,10,12	4.378±0.511^{b*}	2.907±0.271^{a*}	3.638±0.425^{ab*}	2.360±0.267^{a*}
	ipt3,5,7	-	-	-	-
IPT5	WT	1.000±0.086^{b}	0.718±0.084^{a}	1.035±0.119^{b}	0.971±0.096^{b}
	ahk2,3	2.278±0.321^{b*}	1.273±0.139^{a*}	2.046±0.255^{b*}	1.358±0.144^{a}
	arr1,10,12	1.221±0.111^{a}	0.789±0.088^{b}	1.096±0.131^{a}	0.725±0.074^{b}
	ipt3,5,7	-	-	-	-
LOG7	WT	1.000±0.126^{c}	0.220±0.030^{b}	0.872±0.091^{c}	0.104±0.013^{a}
	ahk2,3	0.245±0.019^{b*}	0.102±0.011^{a*}	0.222±0.027^{b*}	0.115±0.014^{a}
	arr1,10,12	0.184±0.021^{ab*}	0.240±0.23^{b}	0.228±0.025^{b*}	0.129±0.008^{a}
	ipt3,5,7	0.215±0.023^{c*}	0.089±0.07^{b*}	0.190±0.020^{c*}	0.058±0.006^{a*}
CKX3	WT	1.000±0.098^{b}	0.791±0.086^{b}	1.123±0.135^{b}	0.354±0.047^{a}
	ahk2,3	0.052±0.006^{b*}	0.018±0.002^{a*}	0.047±0.06^{b*}	0.021±0.003^{a*}
	arr1,10,12	0.142±0.019^{b*}	0.058±0.017^{a*}	0.052±0.006^{a*}	0.050±0.006^{a*}
	ipt3,5,7	1.711±0.166^{c*}	0.269±0.021^{b*}	0.195±0.033^{b*}	0.116±0.012^{a*}
CKX5	WT	1.000±0.102^{a}	1.019±0.084^{a}	0.826±0.084^{a}	0.928±0.093^{a}
	ahk2,3	0.442±0.051^{b*}	0.239±0.025^{a*}	0.394±0.038^{b*}	0.336±0.037^{ab*}
	arr1,10,12	0.263±0.027^{a*}	0.616±0.063^{b*}	0.252±0.025^{a*}	0.247±0.022^{a*}
	ipt3,5,7	1.401±0.137^{ab}	1.470±0.161^{ab*}	1.886±0.235^{b*}	1.237±0.136^{a}
AHK2	WT	1.000±0.098^{c}	0.277±0.033^{a}	1.108±0.124^{c}	0.565±0.066^{b}
	ahk2,3	-	-	-	-
	arr1,10,12	0.501±0.062^{b*}	0.382±0.042^{a}	0.441±0.042^{ab*}	0.391±0.046^{ab*}
	ipt3,5,7	1.316±0.147^{c}	0.236±0.026^{a}	0.582±0.067^{b*}	0.222±0.028^{a*}
AHK3	WT	1.000±0.076^{b}	0.709±0.064^{a*}	1.001±0.085^{b}	0.640±0.052^{a}
	ahk2,3	-	-	-	-
	arr1,10,12	0.514±0.055^{a*}	0.462±0.050^{a*}	0.400±0.048^{a*}	0.459±0.045^{a}
	ipt3,5,7	0.885±0.093^{b}	0.561±0.050^{a}	0.586±0.063^{a*}	0.529±0.054^{a}
AHK4	WT	1.000±0.091^{a}	4.408±0.459^{c}	1.125±0.098^{a}	2.401±0.261^{b}
	ahk2,3	1.409±0.124^{a}	1.654±0.171^{a*}	1.248±0.102^{a}	1.352±0.099^{a*}
	arr1,10,12	0.919±0.085^{a}	1.003±0.103^{a*}	0.726±0.080^{a}	0.753±0.078^{a*}
	ipt3,5,7	0.191±0.016^{a*}	0.877±0.061^{c*}	0.398±0.041^{b*}	0.895±0.092^{c*}

ARR1	WT	1.000±0.079[c]	0.368±0.042[a]	0.916±0.078[c]	0.629±0.072[b]
	ahk2,3	0.905±0.097[b]	0.390±0.047[a]	0.986±0.091[b]	0.405±0.047[a]
	arr1,10,12	-	-	-	-
	ipt3,5,7	0.345±0.040[a*]	0.300±0.032[a]	0.691±0.081[b]	0.275±0.030[a*]
ARR4	WT	1.000±0.088[c]	0.160±0.019[a]	0.850±0.059[c]	0.448±0.021[b]
	ahk2,3	0.449±0.051[a*]	0.503±0.062[a*]	0.491±0.053[a*]	0.391±0.044[a]
	arr1,10,12	0.411±0.045[b*]	0.125±0.011[a*]	0.327±0.036[b*]	0.093±0.012[a*]
	ipt3,5,7	0.106±0.018[a*]	0.113±0.009[a*]	0.138±0.013[a*]	0.130±0.015[a*]
ARR5	WT	1.000±0.077[c]	0.186±0.024[a]	0.703±0.050[b]	0.581±0.044[b]
	ahk2,3	0.921±0.091[a]	0.850±0.091[a*]	0.807±0.081[a]	0.730±0.084[a]
	arr1,10,12	0.371±0.040[b*]	0.075±0.007[a*]	0.316±0.038[b*]	0.080±0.012[a*]
	ipt3,5,7	0.447±0.046[c*]	0.080±0.009[a*]	0.136±0.015[b*]	0.148±0.017[b*]
ARR12	WT	1.000±0.093[c]	0.305±0.041[a]	0.999±0.114[c]	0.568±0.061[b]
	ahk2,3	0.912±0.090[b]	0.409±0.048[a]	0.969±0.090[b]	0.445±0.043[a]
	arr1,10,12	-	-	-	-
	ipt3,5,7	0.232±0.019[b*]	0.280±0.032[a]	0.429±0.046[b*]	0.228±0.024[b*]
CRF6	WT	1.000±0.114[a]	2.214±0.246[b]	0.982±0.086[a]	1.070±0.098[a]
	ahk2,3	1.401±0.165[a]	3.123±0.325[b]	1.352±0.147[a]	3.574±0.277[b*]
	arr1,10,12	0.842±0.094[a]	3.368±0.434[b]	1.124±0.134[a]	4.175±0.422[b*]
	ipt3,5,7	0.228±0.031[a*]	2.721±0.223[c]	0.811±0.099[b]	4.595±0.547[d*]
