# Supplementary material for: Cytokinin Modulates Responses to Phytomelatonin in Arabidopsis thaliana under High Light Stress
Source: Int J Mol Sci. 2023 Jan 1;24(1):738. doi: 10.3390/ijms24010738 (PMC9821067; doi:10.3390/ijms24010738)
Supplement: Supplementary file 1 [file ijms-24-00738-s001.zip › Table S2.pdf]

Table S2. List of primers used for RT-qPCR.

Gene name	Locus	Forward primer (5′-3′)	Reverse primer (5′-3′)
SNAT1	AT1G32070	TAC GCA ACT TGT GGA ACC ACC T	ATC ATA AAC ATC AAT CTC ACC ACC A
ASMT	AT4G35160	GCAAAGAAGCGGTCCC TCCAA	GTCCGTTCTTTGCCT GTGCTTGT
COMT	AT5G54160	GGA GTG ACG AAC ATT GCG T	TCT CGG TTC GTT CTT TGC CT
CAND2	AT3G05010	ATG CGA GTG CTC AGC GAG AT	TTA TTC CCA ATC AGC GTC GAA GAA T
GPA1	AT2G26300	CGT TTG CGA GTG GTT CAG AGA T	CCA AAG CCG TCG TCC TGT AGA T
ELIP2	AT4G14690	ACG GGA GAC TAG CAA TGG TT	CCT AGA AAC CAC CCG ACA CC
psbD	AtCg00270	GGA TGA CTG GTT ACG GAG GG	GGT TGT ACC TGT GAA CCA ACC
rbcL	AtCg00490	CGG GTA CAT GCG AAG AAA TGA	TCT CGG TCA AAG CAG GCA TA
IPT3	AT3G63110	AGT GGA ATG GTT GAG GGA GTC	CAA GAA CTGCTC GTT CCT GA
IPT5	AT5G19040	AGT TAC AGC GAT GAC CAC CA	GGC AGA GAT CTC CGG TAG G
LOG7	AT5G06300	CGC TGT TAA CGT TTA TTG ATA AGG C	TTA TCT CAT CAA ACT CCG GTT CA
CKX3	AT5G56970	TCT CAA TAC ACA GTC AAC GAG GA	TCG TAC ATA AAC CCT CTT ACA TGG
CKX5	AT1G75450	CCA TGG TCC TCA AAT TAG TAA CG	TCT GAG CAT CTC ATC ACC TCT C
AHK2	AT5G35750	GTC TAT AAC TTG TGA GCT CTT GAA TC	GCT CGT GTC ATA GAC AGC AAA GGT C
AHK3	AT1G27320	GCA TCG GAG CTT TGA ACC AT	GGA TAT GGA TGG TCC GAC TTG
AHK4	AT1G27320	ATT CTA GCG ATG ACT GCG GA	CGA CGA AGG TGA GAT AGG ATT
ARR1	AT3G16857	ACA ACG GAA TGC TGA TGC CTC T	CAC TCT GCT GCT CTC GGG GAT
ARR4	AT1G10470	CGG AGA ATG TAT TGA CCA GAA TC	AGA AAT CTT GAG CAC CTT CCT C
ARR5	AT3G48100	CTA CTC GCA GCT AAA ACGC	GCC GAA AGA ATC AGG ACA
ARR12	AT2G25180	GTT TCC ATC ACC GCC CAA TC	CCG AAG GAG TAT TGA GTC TGC CA
CRF6	At4g23750	AAT CCG TTA CTG CTT CTT CCT CC	AGA CAC CGG AGA GCA GAG ACA A
UBQ10	At4G05320	GCG TCT TCG TGG TGG TTT CTA A	GAA AGA GAT AAC AGG AAC GGA AAC A

Sequence data from this article can be found in the National Center for Biotechnology Information https://www.ncbi.nlm.nih.gov/
