# Supplementary material for: Cytokinin Modulates Responses to Phytomelatonin in Arabidopsis thaliana under High Light Stress
Source: Int J Mol Sci. 2023 Jan 1;24(1):738. doi: 10.3390/ijms24010738 (PMC9821067; doi:10.3390/ijms24010738)
Supplement: Supplementary file 1 [file ijms-24-00738-s001.zip › Table S3.pdf]

Table S3. (**a**) Influence of photostress of different duration and melatonin on the parameter Fv/Fm in the wild type; (**b**) Influence of different melatonin pretreatment times on its protective properties under photostress (TBARs). Different letters denote statistically significant differences between variants within the same genotype at $p < 0.05$ (ANOVA with post hoc Tukey's multiple-comparison test)

(a)	
**Conditions**	**Values**
3 h	
Control	0.809±0.007 [a]
Stress	0.625±0.014 [b]
Control + melatonin	0.810±0.011 [a]
Stress + melatonin	0.673±0.019 [c]
9 h	
Control	0.812±0.015 [a]
Stress	0.602±0.018 [b]
Control + melatonin	0.806±0.009 [a]
Stress + melatonin	0.635±0.020 [b]
24 h	
Control	0.815±0.014 [a]
Stress	0.427±0.017 [b]
Control + melatonin	0.808±0.012 [a]
Stress + melatonin	0.651±0.024 [c]
(b)	
**Conditions**	**Values**
24 h	
Control	2.356±0.128[a]
Stress	10.011±0.715[b]
Control + melatonin	2.504±0.089[a]
Stress + melatonin	8.011±0.077[c]
72 h	
Control	2.772±0.144[a]
Stress	9.518±0.807[b]
Control + melatonin	2.824±0.196[a]
Stress + melatonin	5.877±0.442[c]
